# Tailoring mSiO_2_-SmCo_x_ nanoplatforms for magnetic/photothermal effect-induced hyperthermia therapy

**DOI:** 10.3389/fbioe.2023.1249775

**Published:** 2023-07-27

**Authors:** Xinqiang Liang, Wenting Xu, Siyi Li, Mekhrdod S. Kurboniyon, Kunying Huang, Guilan Xu, Wene Wei, Shufang Ning, Litu Zhang, Chen Wang

**Affiliations:** ^1^ Department of Research, Guangxi Medical University Cancer Hospital, Nanning, China; ^2^ College of Material Sciences and Chemical Engineering, Harbin Engineering University, Harbin, China; ^3^ National Academy of Sciences of Tajikistan, Dushanbe, Tajikistan

**Keywords:** magnetic effect, photothermal effect, permanent magnet, mSiO_2_, tumor therapy

## Abstract

Hyperthermia therapy is a hotspot because of its minimally invasive treatment process and strong targeting effect. Herein, a synergistic magnetic and photothermal therapeutic nanoplatform is rationally constructed. The well-dispersive mSiO_2_-SmCo_x_ nanoparticles (NPs) were synthesized through a one-step procedure with the regulated theoretical molar ratio of Sm/Co among 1:1, 1:2, and 1:4 for controlling the dispersion and magnetism properties of SmCo_x_ NPs *in situ* growth in the pore structure of mesoporous SiO_2_ (mSiO_2_), where mSiO_2_ with diverse porous structures and high specific surface areas serving for locating the permanent magnetic SmCo_x_ NPs. The mSiO_2_-SmCo_x_ (Sm/Co = 1:2) NPs with highly dispersed and uniform morphology has an average diameter of ∼73.08 nm. The photothermal conversion efficiency of mSiO_2_-SmCo_x_ (Sm/Co = 1:2) NPs was determined to be nearly 41%. The further *in vitro* and *in vivo* anti-tumor evaluation of mSiO_2_-SmCo_x_ (Sm/Co = 1:2) NPs present promising potentials for hyperthermia-induced tumor therapy due to magnetic and photothermal effects.

## 1 Introduction

Malignant tumors seriously infringe on public health and are the diseases with the highest fatality rate (Tan et al., 2022; Zhang et al., 2022). Abnormal growth and reproduction of tumor cells may spread to tissues and organs throughout the body through the blood and lymphatic system at any time, bringing serious consequences. In recent years, with the improvement of the clinical medical level, remarkable progress has been made in tumor diagnosis and treatment. However, due to the rapid growth and reproduction of tumor cells and ease to spread, the treatment is still a huge challenge. On the other hand, currently widely used traditional treatment methods have great limitations and side effects. Thus, researchers are trying to find new treatments with less toxic side effects and better results. The prosperous development of nanotechnology has provided a new approach for cancer therapeutics, reduced the side effects, and enhanced the targeting efficiency of anti-tumor drugs (Cai et al., 2019; Chang et al., 2021; Li et al., 2021; Xu and Pu, 2021; Lv et al., 2022; Cao et al., 2023; Li et al., 2023). Significantly, tumor hyperthermia, as a new type of tumor adjuvant therapy, has achieved significant advances (Lv et al., 2017; Liu et al., 2021; Uson et al., 2021; Yu et al., 2021; Chung et al., 2022; Wang et al., 2022). Among them, magnetic hyperthermia therapy and photothermal therapy are becoming hot spots due to their advantages of minimally invasive treatment processes and strong targeting effect (Wu et al., 2019; Zhu et al., 2019; Xu et al., 2020a; Li et al., 2020; Zhang et al., 2020; Zhang X. et al., 2021; Castellanos-Rubio et al., 2021; Wang and Hou, 2021).

Tumor hyperthermia is defined as the method of heating the tumor area to 41–46°C for treatment, while thermal ablation of the tumor refers to the method of heating the tumor area to more than 56°C to make the tumor tissue coagulated and necroti. (Murugan et al., 2019; Lima et al., 2021; Lu and Wang, 2021; Xu and Pu, 2021). Tumor cells have poor heat resistance and are prone to apoptosis and necrosis at 40–48°C, while normal cells and tissues are not affected. Traditional hyperthermia has certain side effects, so it is only used as an auxiliary means of radiotherapy and chemotherapy. In recent years, nanotechnology-induced tumor hyperthermia as a new hyperthermia method has attracted wide attention. Tian *et al.* reported a near-infrared (NIR)-triggered theranostic nanoplatform (GA-PB@MONs@LA) for synergistic photothermal therapy and enhanced Fenton nanocatalytic therapy against hypoxic tumors (Tian et al., 2022). Magnetic hyperthermia therapy uses the high temperature generated by the magnetic thermal effect, where the magnetic thermal materials in the high-frequency alternating magnetic field (AMF) generate heat to eliminate tumor cells (Beola et al., 2020; Fotukian et al., 2020; Idoia et al., 2020; Qian et al., 2020; Xie et al., 2020; Zhao et al., 2020). Compared with traditional therapies, magnetic hyperthermia therapy is not limited by the depth of treatment and is a non-invasive treatment with strong specificity and targeted effect, which can be used as a sensitizer in combination with chemotherapy, radiation therapy, immunotherapy, and gene therapy to achieve synergistic results. Photothermal therapy uses photothermal agents to transfer light energy into heat energy under light irradiation and release a large amount of heat to ablate tumor cells (Fan et al., 2018; Dalila et al., 2019; Wang et al., 2019; Xu et al., 2019; Zhou et al., 2019; Shi et al., 2021). The high temperature will not only damage the cell membrane of tumor cells and denature proteins but also inhibit the replication of DNA for eliminating tumor cells. Photothermal therapy has the advantages of minimal invasion, low toxicity, and side effects, and high photothermal conversion efficiency, which has great development potential in the field of tumor theranostics (Zeng et al., 2018; Cai et al., 2019; Chen et al., 2019; Deng et al., 2019; Lu et al., 2023). Thus, constructing the “all-in-one” nanoplatforms with both magnetic and photothermal effects for high efficacy of tumor hyperthermia is interesting.

Magnetic nanomaterials with high Curie temperature, high coercivity, good magnetic thermal properties, etc., have a wide range of applications in tumor diagnosis and treatment (Xu et al., 2020b; Wang et al., 2020; Xie et al., 2020). Firstly, they are able to be employed as contrast agents for bio-imaging. Secondly, magnetic nanomaterials gathered inside tumor cells can convert electromagnetic energy into heat energy in the high-frequency AMF and release heat, thus causing tumor cell apoptosis or tumor tissue necrosis (Umut et al., 2019; Chan et al., 2020; Chandrasekharan et al., 2020). Thirdly, the modified magnetic nanomaterials can achieve specific delivery of drugs under the traction of AMF. The magnetic nanomaterials studied at present are mainly iron-based nanomaterials. Interestingly, nanocrystalline permanent magnet materials also show excellent magnetic properties and gradually become a research hotspot in the therapeutic field (Zhang Y. et al., 2021; Wang and Hou, 2021; Chung et al., 2022; Wang et al., 2022). Magnetic materials can be divided into three categories, namely, hard magnetism, semi-hard magnetism, and soft magnetism. The hard magnetic material with high coercivity is not easy to demagnetize after magnetization. After removing the external magnetic field, it can still maintain strong magnetic materials, which is also known as permanent magnet material. So far, rare Earth permanent magnets have gone through three generations, namely, SmCo_5_, Sm_2_Co_17_, and Nd-Fe-B permanent magnet materials. However, controlling the size of permanent magnetic materials in the nanoscale is an important prerequisite for their development in the medical field. Surprisingly, mesoporous silica with diverse porous structures and high specific surface areas can be utilized to locate the permanent magnet nanomaterial separately.

Herein, the well-dispersive mSiO_2_-SmCo_x_ NPs were synthesized through a one-step procedure for magnetic/photothermal effect-induced hyperthermia therapy. The theoretical molar ratio of Sm/Co was systematically regulated among 1:1, 1:2, and 1:4 for controlling the dispersion and magnetism properties of SmCo_x_ NPs *in situ* growth in the pore structure of mesoporous SiO_2_ (mSiO_2_). The mSiO_2_-SmCo_x_ (Sm/Co = 1:2) NPs with highly dispersed and uniform morphology has an average diameter of ∼73.08 nm, and the photothermal conversion efficiency was determined to be nearly 41%. The further *in vitro* and *in vivo* anti-tumor evaluation of mSiO_2_-SmCo_x_ (Sm/Co = 1:2) NPs demonstrated promising potentials for hyperthermia-induced tumor therapy due to magnetic and photothermal effects.

## 2 Experimental sections

### 2.1 Materials

Samarium(III) 2,4-pentanedionate hydrate (Sm(acac)_3_, 98%), cobalt(III) acetylacetonate (Co(acac)_3_, 98%), acetic acid (CH_3_COOH), polyvinyl pyrrolidone (PVP, 13K), hydrochloric acid (HCl, 30 wt%), hydrogen peroxide (H_2_O_2_, 30 wt%), hexadecyl trimethyl ammonium chloride (CTAC), triethylene glycol (TEG), and tetraethylorthosilane (TEOS) were purchased from Aladdin Reagent Co. Ltd. propidium iodide (PI, 98%), calcein-acetoxymethyl ester (AM, 97%), 3-(4,5-dimethyl-2-thiazolyl)-2,5-diphenyl-2-H-tetrazolium bromide (MTT, 97%), and fluorescein isothiocyanate (FITC) were obtained from Beyotime Biotechnology Co., Ltd.

### 2.2 Synthesis process

For mSiO_2_-SmCo_x_ NPs, the molar ratio of Sm/Co ions was adjusted as 1:1, 1:2, and 1:4. A fixed amount of Sm(NO_3_)_3_ and the corresponding amount of Co(NO_3_), as-synthesized mesoporous silicon, and 25 mL of PVP solution (1g mL^–1^, in TEG) were mixed through magnetic stirring. Under the vacuum condition, the mixture was heated to 120°C. Later, a special amount of acetic acid was added and continuously stirred (300 rpm) for 20 min. Next, the mixture was slowly heated to 260°C at a constant speed (5°C min^–1^) under the protection of N_2_ atmosphere and reacted for another 2 h. After naturally cooling to 25°C, the product was pretreated by centrifugation treatment (12000 rpm, 10 min) and washed with cyclohexane and ethanol for four times. The mSiO_2_-SmCo_x_ NPs were then obtained after drying overnight (80°C).

### 2.3 Characterizations

The transmission electron microscopy (TEM, containing EDS and mapping) images were measured on the Tecnai T20 microscope with an operating voltage of 200 kV. The XRD patterns were explored on a DMAX-2400 diffractometer with Cu Kα radiation under 40 kV. X-ray photoelectron spectroscopy (XPS) analysis was operated on a Thermo Scientific K-Alpha spectroscope. Inductively coupled plasma optical emission spectrometer (ICP-OES) was obtained by iCAP 6000 series spectrometry. Ultraviolet-Near Infrared (UV-NIR) absorbance value was tested by a TU-1601 spectrophotometer. The fluorescence intensity of cells and stained tissue sections were obtained with confocal irradiation scanning microscopy TCS SP8. Apoptosis data were characterized by flow cytometry.

### 2.4 Magnetic/photothermal properties

Different concentration of mSiO_2_-SmCo_x_ (Sm/Co = 1:2) NPs solution was placed in a 1.5 mL tube, illuminated with 808 nm laser (1.0 W cm^−2^) for 800 s or imposed on AMF condition. The mSiO_2_-SmCo_x_ (Sm/Co = 1:2) NPs solution was placed in the middle of the coils of an in-house-built magnetic hyperthermia device (coil diameter: 10 cm, frequency: 513 kHz, output power: 8 kW, output current: 28.2 A, output voltage: 361 V). Then, the photograph was captured by an Infrared thermal camera. Besides, the PBS solution as control was irradiated in the same way.

### 2.5 *In vitro* experiments

The cell lines (L929 and 4T1) present in this study were obtained from FDCC (Ruilu in Shanghai, China). The cell strains were cultivated at 37°C under 5% CO_2_. Firstly. The confocal laser scanning microscope (CLSM) images were measured to investigate the cell phagocytosis in 4T1 cells. Followed by setting in a cell culture dish with 28 mm cover glass, The 4T1 cells (1 × 10^5^ per well) were incubated overnight. Then, DMEM solution loading FITC-labeled mSiO_2_-SmCo_x_ (Sm/Co = 1:2) NPs (1 mL, 50 μg mL^–1^) were added in different time nodes (1, 2, and 3 h). Afterward, an MTT assay was processed to estimate the biocompatibility and cytotoxicity of mSiO_2_-SmCo_x_ (Sm/Co = 1:2) NPs using 4T1 and L929 cells with different concentrations and conditions. With the injection of MTT solution (200 μL, 1 mg mL^–1^) into 4T1 cells for 4 h, the corresponding absorbance was measured to figure out the cell viability. As the above cell treatment, the 4T1 cells were subjected to live/dead cell staining and cultured for 12 h. Then, the cells in each well were treated with different groups and stained by calcein-AM/PI (4 μM and 8 Μm, respectively) for CLSM observation. For quantitative analysis of cell death, the collecting cells were treated with an annexin V-FITC/PI dual-staining apoptosis detection kit for flow cytometry examination.

### 2.6 *In vivo* experiments

The animal experiments were approved by the Ethics Committee of Guangxi Medical University Cancer Hospital, and have been implemented in accordance with its protocol. All BALB/c female mice derived from Beijing Vital River Laboratory Animal Technology Co., Ltd. (About 4 weeks old, 1100111084356) were injected with 4T1 cells (2 × 10^6^, fixing at the right subcutaneous back). When the tumor-bearing volume increased to 30 mm^3^, the mice were intravenously administered with mSiO_2_-SmCo_x_ (Sm/Co = 1:2) NPs (*n* = 3, 20 mg kg^−1^). After post-injection at 1, 3, 6, 12, and 24 h, the five main organs (heart, kidney, liver, lung, and spleen) of the executed mice were extracted for Sm ions biodistribution evaluation using ICP-OES, and the tumor weight was recorded. Twenty mice were randomly divided into four groups (4T1 tumor-bearing, *n* = 5): 1) control (PBS), 2) mSiO_2_-SmCo_x_ (Sm/Co = 1:2) NPs, 3) mSiO_2_-SmCo_x_ (Sm/Co = 1:2) NPs + NIR (1.0 W cm^−2^, 10 min), and 4) mSiO_2_-SmCo_x_ (Sm/Co = 1:2) NPs + NIR (1.0 W cm^−2^, 10 min) + M (AFM, in-house-built magnetic hyperthermia device with coil diameter: 10 cm, frequency: 513 kHz, output power: 8 kW, output current: 28.2 A, output voltage: 361 V). mSiO_2_-SmCo_x_ (Sm/Co = 1:2) NPs were injected intravenously into each group on 1, 7, and 14 days. All the samples were exposed to near-infrared (NIR) irradiation or magnetic conditions after 6 h intravenously injection. The body weights and tumor progression were evaluated every 2 days. The tumor volume (mm^3^) was measured by the equation, *V* = *lw* ^2^/2, where *l* (*w)* represents the longer (shorter) dimension of the tumor, respectively. After the treatment procedure, the tumor and major organs (heart, kidney, liver, lung, and spleen) were collated and stained for CLSM observation.

### 2.7 Statistical analysis

The results are presented as mean ± S.D. Error bars are dependent on the standard errors of the mean (*n* = 5). Statistical analysis is presented by the Student’s two-sided *t*-test. *p < 0.05, **p < 0.01, or ***p < 0.001.

## 3 Results and discussion

### 3.1 Synthesis and characterization

**FIGURE 1:**
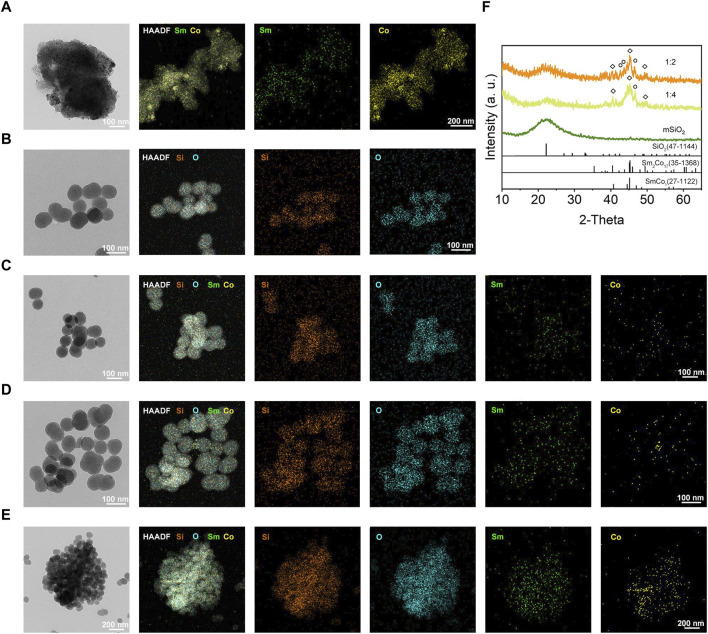
**(A–E)** TEM, HAADF-TEM, and element mapping (Si, O, Sm, and Co) of SmCo_x_, mSiO_2_, and mSiO_2_-SmCo_x_ (Sm/Co = 1:1, 1:2, and 1:4) NPs. **(F)** XRD patterns of mSiO_2_ and mSiO_2_-SmCo_x_ (Sm/Co = 1:2 and 1:4).

**FIGURE 2:**
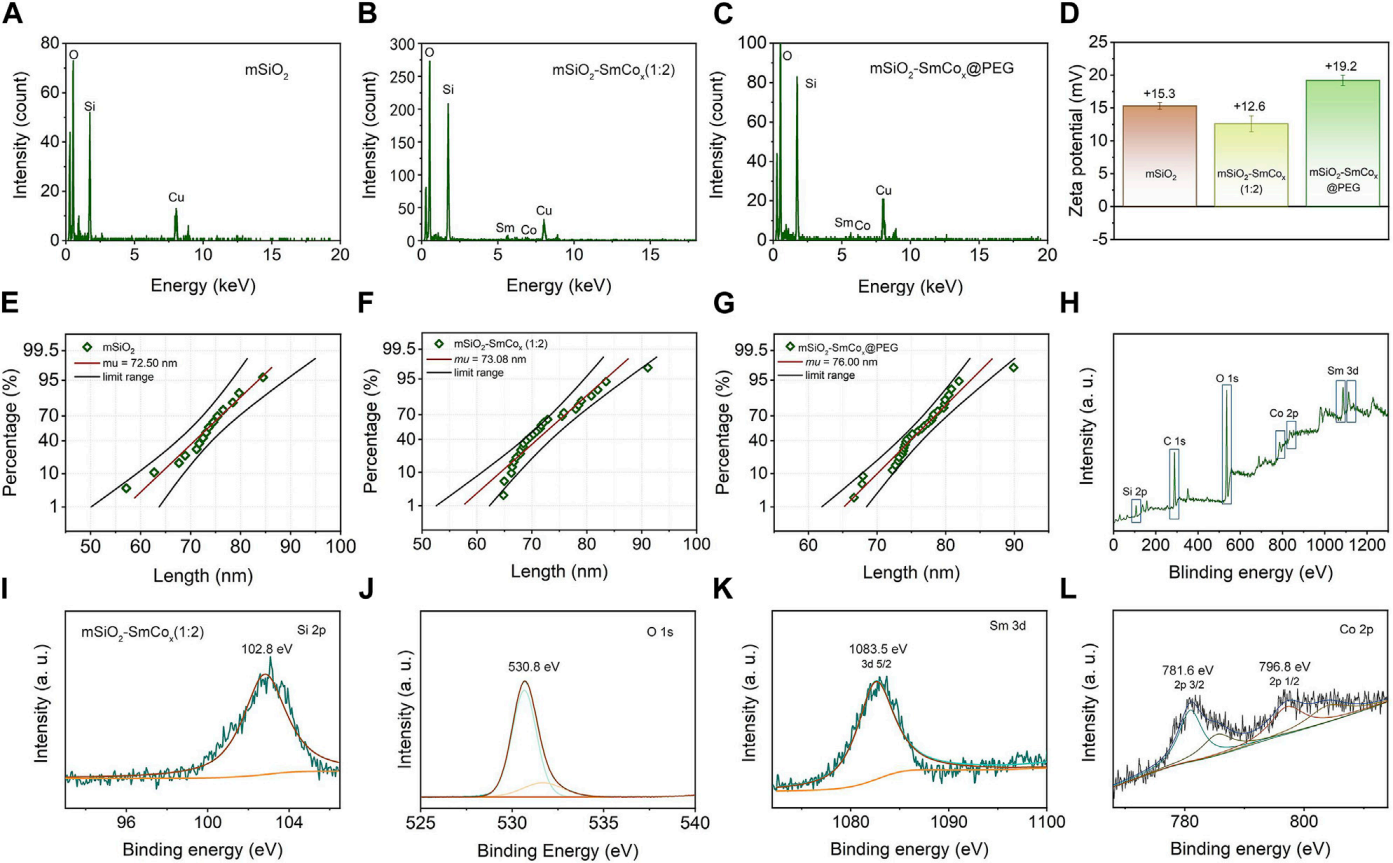
**(A–C)** EDS spectra of mSiO_2_ and mSiO_2_-SmCo_x_ (Sm/Co = 1:2 and 1:4) NPs. **(D)** Zeta potential of mSiO_2_, mSiO_2_-SmCo_x_ (Sm/Co = 1:2) NPs, and PEG-modified mSiO_2_-SmCo_x_ (Sm/Co = 1:2) NPs. **(E–G)** Size distribution of mSiO_2_, mSiO_2_-SmCo_x_ (Sm/Co = 1:2) NPs, and PEG-modified mSiO_2_-SmCo_x_ (Sm/Co = 1:2) NPs. **(H)** XPS spectrum of mSiO_2_-SmCo_x_ (Sm/Co = 1:4) NPs. **(I–L)** High-resolution XPS spectra of mSiO_2_-SmCo_x_ (Sm/Co = 1:2) NPs: Si 2p, O 1s, Sm 3d, and Co 2p.

The mSiO_2_-SmCo_x_ NPs were simply synthesized through a one-step procedure, where the SmCo_x_ NPs were *in situ* grown in the pore structure of mSiO_2_. The theoretical molar ratio of Sm/Co was regulated among 1:1, 1:2, and 1:4 for controlling the dispersion and magnetism properties of mSiO_2_-SmCo_x_ NPs. As exhibited in Figures 1A–E, the TEM images display the nanostructure of SmCo_x_ NPs, mSiO_2_, and mSiO_2_-SmCo_x_ NPs (Sm/Co = 1:1, 1:2, and 1:4). For SmCo_x_ NPs without mSiO_2_ (Figure 1A), the free*-*grown SmCo_x_ NPs are exhibited in the aggregated performance due to the magnetic properties of SmCo_x_ NPs, which are excluded for further bio-application. The pure mSiO_2_ TEM image in Figure 1B demonstrates the well-dispersive manners and the large amount of pore structure. For mSiO_2_-SmCo_x_ (Sm/Co = 1:1 and 1:2) NPs (Figures 1C, D), a uniform spherical morphology with highly monodisperse is observed. The corresponding Sm, Co, Si, and O elements mappings demonstrate the loading and separation functionalities of mSiO_2_ nanocarriers and also demonstrate the collective of Co and Sm elements. For mSiO_2_-SmCo_x_ (Sm/Co = 1:4) NPs (Figure 1E), the SmCo_x_ NPs in the pore structure of mSiO_2_ show an enhanced aggregated manner. The X-ray diffraction (XRD) patterns of mSiO_2_ and mSiO_2_-SmCo_x_ (Sm/Co = 1:2 and 1:4) NPs are shown in Figure 1F, from which the characteristic diffraction peaks could be well indexed to the SmCo_5_ (JCPDS No. 35–1368) and mSiO_2_ (JCPDS No. 47–1144), respectively, demonstrating the successful formation of magnetic nanoparticles of SmCo_5_. The elemental composition of mSiO_2_ and mSiO_2_-SmCo_x_ (Sm/Co = 1:2 and 1:4) NPs also reveals by energy-dispersive spectroscopy (EDS) mapping images (Figures 2A–C). The zeta potential is explored for mSiO_2_, mSiO_2_-SmCo_x_ (Sm/Co = 1:2) NPs, and PEG-modified mSiO_2_-SmCo_x_ (Sm/Co = 1:2) NPs, changing from 15.3 eV, 12.6 eV, to 19.2 eV, illustrating the good biocompatibility (Figure 2D). The particle size distribution patterns indicate that the mSiO_2_, mSiO_2_-SmCo_x_ (Sm/Co = 1:2) NPs, and PEG-modified mSiO_2_-SmCo_x_ (Sm/Co = 1:2) NPs with high dispersity and uniform morphology have an average diameter of 72.50 73.08, and 76.00 nm, respectively (Figures 2E–G). Furtherly, the element composition is again demonstrated by XPS. The full-scan XPS confirms the existence of Sm 3d, Co 2p, Si 2p, and O 1s in mSiO_2_-SmCo_x_ (Sm/Co = 1:2) NPs (Figure 2H). The binding energy peaks of Si 2p are given in Figure 2I for the form of Si in mSiO_2_. Through peak separation. The high-resolution XPS spectrum of O (Figure 2J) shows the banding energy of 530.8 eV. The high-resolution spectra of Sm 3d at a peak of 1083.5 eV are attributed to Sm 3d5/2 (Figure 1K). From the high-resolution of Co 3d (Figure 2L), two peaks at 781.6 eV and 796.8 eV are attributed to Co 2p_3/2_ and Co 2p_1/2_, respectively. The above structure and morphology analysis demonstrate that the mSiO_2_-SmCo_x_ NPs with proper Sm/Co molar ratio equip with promising potentials for further bio-application.

### 3.2 Magnetic/photothermal effects evaluation

**FIGURE 3:**
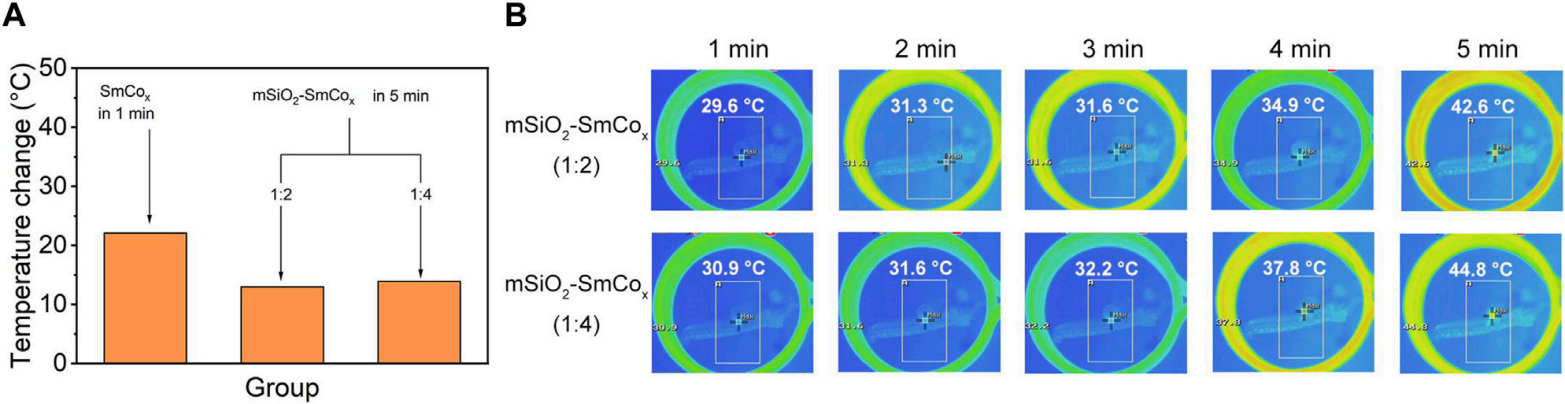
**(A)** Temperature increases of SmCo_x_ (within 1 min) and mSiO_2_-SmCo_x_ (Sm/Co = 1:2 and 1:4) NPs (within 5 min) under AMF. **(B)** Infrared thermal images of mSiO_2_-SmCo_x_ (Sm/Co = 1:2 and 1:4) NPs for 1–5 min.

Magnetic hyperthermia therapy and photothermal therapy are becoming widespread hotspots due to their advantages of minimally invasive treatment processes and strong targeting effect. Herein, the magnetic and photothermal effects were explored in detail. Compared with optical, acoustic, and electrical fields, the magnetic field shows properties with large force output, high precision, and especially deep tissue penetration. Firstly, the hyperthermia induced by SmCo_x_ NPs and mSiO_2_-SmCo_x_ (Sm/Co = 1:2 and 1:4) NPs under AFM was characterized. The pure SmCo_x_ NPs group shows the highest temperature increase compared with the mSiO_2_-SmCo_x_ (Sm/Co = 1:2 and 1:4) NPs. Among the mSiO_2_-SmCo_x_ NPs, the hyperthermia-induced performance of mSiO_2_-SmCo_x_ (Sm/Co = 1:4) NPs is better than mSiO_2_-SmCo_x_ (Sm/Co = 1:2) NPs (Figure 3A). The corresponding infrared thermal images of mSiO_2_-SmCo_x_ (Sm/Co = 1:2 and 1:4) NPs directly demonstrate the above result (Figure 3B). However, the aggregated performance of mSiO_2_-SmCo_x_ (Sm/Co = 1:4) NPs is not suitable for bio-application. Thus, according to the structural characterization and magnetic effect property, the mSiO_2_-SmCo_x_ (Sm/Co = 1:2) NPs are chosen for further investigation.

**FIGURE 4:**
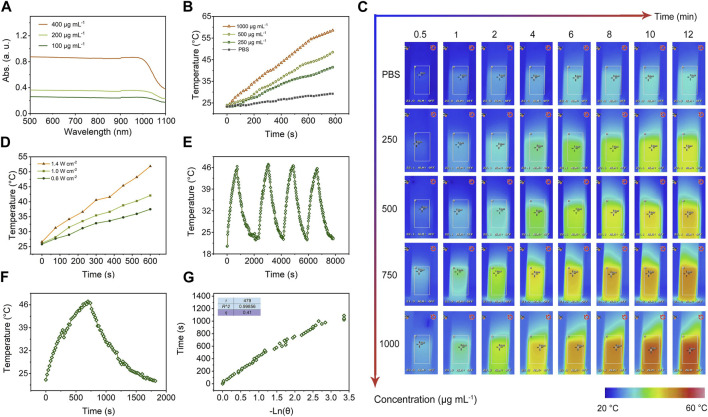
**(A)** UV-NIR spectra of aqueous suspensions of dispersed mSiO_2_-SmCo_x_ (Sm/Co = 1:2) NPs at varied concentrations (100, 200, and 400 μg mL^−1^). **(B)** Photothermal heating curves of dispersed mSiO_2_-SmCo_x_ (Sm/Co = 1:2) NPs at varied concentrations (0, 200, 500, and 1000 μg mL^−1^) under 808 nm laser with a power density of 1.0 W cm^−2^. **(C)** Infrared thermal images of PBS (control) and dispersed mSiO_2_-SmCo_x_ (Sm/Co = 1:2) NPs at varied concentrations (250, 500, 750, and 1000 μg mL^−1^) under irradiation by an 808 nm laser with a power density of 1.0 W cm^−2^ for 12 min. **(D)** Photothermal heating curves of 500 μg mL^−1^ dispersed mSiO_2_-SmCo_x_ (Sm/Co = 1:2) NPs at varied power densities (0.6, 1.0, and 1.4 W cm^−2^). **(E)** Recycling-heating profiles of 500 μg mL^−1^ dispersed mSiO_2_-SmCo_x_ (Sm/Co = 1:2) NPs aqueous solution after 808 nm laser irradiation at 1.0 W cm^−2^ for four laser on/off cycles. **(F)** Heating and cooling curves of an aqueous dispersion of 500 μg mL^−1^ dispersed mSiO_2_-SmCo_x_ (Sm/Co = 1:2) NPs aqueous solution after 808 nm laser irradiation at 1.0 W cm^−2^. **(G)** Photothermal conversion efficiency at 808 nm. The time constant (*τ*_s_) for heat transfer from the system was determined to be *τ*_s_ = 479 s.

Lately, the hyperthermia induced by NIR irradiation was measured with photothermal conversion efficiency (*η*) to determine the light-heat conversion performance of mSiO_2_-SmCo_x_ (Sm/Co = 1:2) NPs. As shown in Figure 4A, the synthesized mSiO_2_-SmCo_x_ (Sm/Co = 1:2) NPs present a broad absorption ranging from ultraviolet (UV) to NIR wavelengths. The UV-NIR spectra of dispersed mSiO_2_-SmCo_x_ (Sm/Co = 1:2) NPs aqueous suspensions at varied concentrations (100, 200, and 400 μg mL^−1^) show distinctive concentration-dependent light absorption. The obvious NIR absorption of mSiO_2_-SmCo_x_ (Sm/Co = 1:2) NPs manifests that they can transfer NIR light into heat, which has great potential in photothermal therapy for killing tumor cells. The photothermal heating curves of dispersed mSiO_2_-SmCo_x_ (Sm/Co = 1:2) NPs at varied concentrations (200, 500, and 1000 μg mL^−1^) under1.0 W cm^−2^ 808 nm NIR irradiation display a significant temperature increase based on their concentration (Figure 4B). After NIR (808 nm, 1.0 W cm^−2^) irradiation for 5 min, the temperature of mSiO_2_-SmCo_x_ NPs aqueous solution (Sm/Co = 1:2, 1000 μg mL^−1^) is increased by 41.2°C. However, the control group which only increase by 2.8°C under the same conditions, further demonstrated the ability of mSiO_2_-SmCo_x_ (Sm/Co = 1:2) NPs for increasing the solution temperature. Moreover, under 808 nm laser (1.0 W cm^−2^, 12 min) irradiation, the corresponding infrared thermal photos of PBS (control) and dispersed mSiO_2_-SmCo_x_ (Sm/Co = 1:2) NPs at varied concentrations (250, 500, 750, and 1000 μg mL^−1^) exhibit a significant temperature increase and color change, confirming the ablation potential for *in vitro* and *in vivo* tumor (Figure 4C). Besides, the heating curves of 500 μg mL^−1^ dispersed mSiO_2_-SmCo_x_ (Sm/Co = 1:2) NPs at varied power densities (0.6, 1.0, and 1.4 W cm^−2^) were also explored and demonstrated the power density-related temperature increase manner of mSiO_2_-SmCo_x_ (Sm/Co = 1:2) NPs (Figure 4D). The capability of photothermal conversion was also estimated. After 808 nm NIR irradiation (1.0 W cm^−2^, four on/off cycles), the recycling-heating profiles of 500 μg mL^−1^ dispersed mSiO_2_-SmCo_x_ (Sm/Co = 1:2) NPs aqueous solution demonstrated good stability of mSiO_2_-SmCo_x_ (Sm/Co = 1:2) NPs (Figure 4E). Subsequently, the heating and cooling curves of an aqueous dispersion of 500 μg mL^−1^ dispersed mSiO_2_-SmCo_x_ (Sm/Co = 1:2) NPs with the same conditions were recorded (Figure 4F). Accordingly, the time constant (τ_s_) and photothermal conversion efficiency (*η*) of mSiO_2_-SmCo_x_ (Sm/Co = 1:2) NPs were calculated to be nearly 479 s and 41%, respectively.

### 3.3 *In vitro* evaluation

**FIGURE 5:**
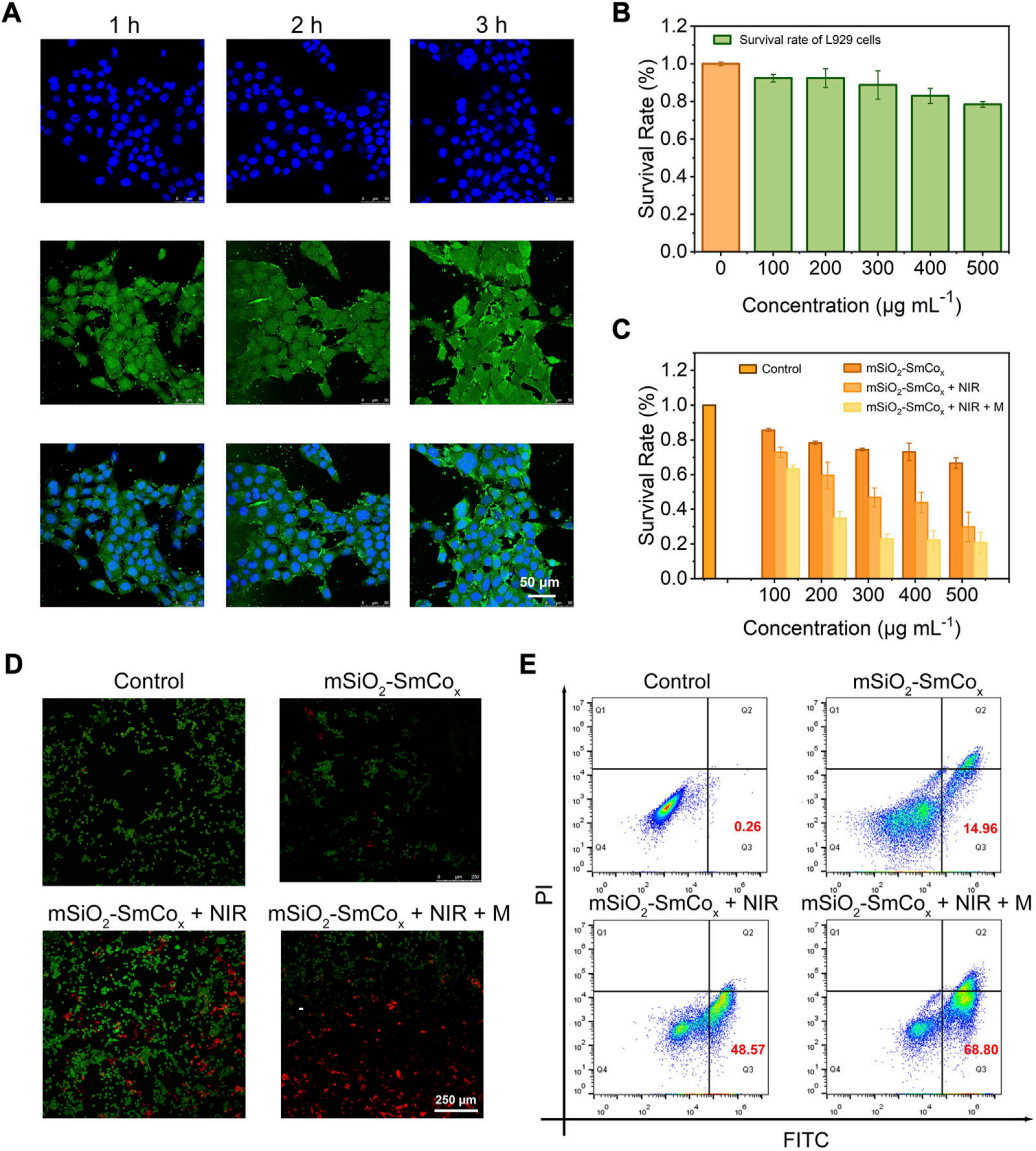
**(A)** CLSM images of 4T1 cells after coincubation with mSiO_2_-SmCo_x_ (Sm/Co = 1:2) NPs for 1, 2, and 3 h **(B)** The survival rate of L919 cells after coincubation with mSiO_2_-SmCo_x_ (Sm/Co = 1:2) NPs under different concentrations (100, 200, 300, 400, and 500 μg mL^–1^). **(C)** The survival rate of 4T1 cells after coincubation with mSiO_2_-SmCo_x_ (Sm/Co = 1:2) NPs, mSiO_2_-SmCo_x_ (Sm/Co = 1:2) NPs + NIR, mSiO_2_-SmCo_x_ (Sm/Co = 1:2) NPs + NIR + M groups under different concentrations (100, 200, 300, 400, and 500 μg mL^–1^). **(D)** AM/PI staining of 4T1 cells after different treatments. **(E)** Flow cytometry results of 4T1 cells after different treatments.

By virtue of the excellent magnetic and photothermal effects of mSiO_2_-SmCo_x_ (Sm/Co = 1:2) NPs, the *in vitro* antitumor efficacy of mSiO_2_-SmCo_x_ (Sm/Co = 1:2) NPs was investigated. Firstly, as the most popular model of NPs being uptaken into cells, the endocytosis manner of mSiO_2_-SmCo_x_ (Sm/Co = 1:2) NPs was investigated (Figure 5A). The FITC-conjugated mSiO_2_-SmCo_x_ (Sm/Co = 1:2) NPs could be uptaken by tumor cells as evidenced by the time-dependent green fluorescence from FITC emission. Then, the biocompatibility and biotoxicity of mSiO_2_-SmCo_x_ (Sm/Co = 1:2) NPs were assessed by standard methyl thiazolyl tetrazolium (MTT) assay using L929 fibroblast normal cells and 4T1 breast cancerous cells. The biocompatibility was assessed after the cultivation of mSiO_2_-SmCo_x_ (Sm/Co = 1:4) NPs against L929 cells with different concentrations (100, 200, 300, 400, and 500 μg mL^−1^). As displayed in Figure 5B, even at high dose levels (500 μg mL^−1^), the survival rate of cells (24 h) is still great high (>90%), demonstrating that the mSiO_2_-SmCo_x_ (Sm/Co = 1:2) NPs exhibit no significant cytotoxicity toward normal cells. However, the *in vitro* biotoxicity of mSiO_2_-SmCo_x_ (Sm/Co = 1:2) NPs towards 4T1 cells with various treatments at different concentrations of mSiO_2_-SmCo_x_ (Sm/Co = 1:2) NPs is discriminative. The group under NIR irradiation alone shows apparent damage against 4T1 cells due to the photothermal effect of mSiO_2_-SmCo_x_ (Sm/Co = 1:2) NPs (Figure 5C). The group treated with mSiO_2_-SmCo_x_ (Sm/Co = 1:2) NPs under NIR irradiation and magnetic condition show the lowest survival rate due to both the photothermal effect and magnetic effect of mSiO_2_-SmCo_x_ (Sm/Co = 1:2) NPs under NIR irradiation and magnetic condition. Furthermore, all the treatment groups represent cytotoxicity in a concentration-correlated manner. As expected, the CLSM images of co-stained AM/PI show the strongest red fluorescence signal under the treatment of mSiO_2_-SmCo_x_ (Sm/Co = 1:2) NPs under NIR irradiation and magnetic condition (Figure 5D), which illustrates the largest number of apoptotic cells. The same cytotoxicity was further confirmed *via* the quantitative flow cytometry analysis (Figure 5E). The apoptotic ratio of the group treated with mSiO_2_-SmCo_x_ (Sm/Co = 1:2) NPs under NIR irradiation and magnetic condition, 68.80% (Q2 + Q3), show a more significant amount than the groups of mSiO_2_-SmCo_x_ (Sm/Co = 1:2) NPs under NIR irradiation (48.57%) and mSiO_2_-SmCo_x_ (Sm/Co = 1:2) NPs (14.96%), demonstrating cytotoxicity could be defined as synergistic photothermal and magnetic effects.

### 3.4 *In vivo* evaluation

**FIGURE 6:**
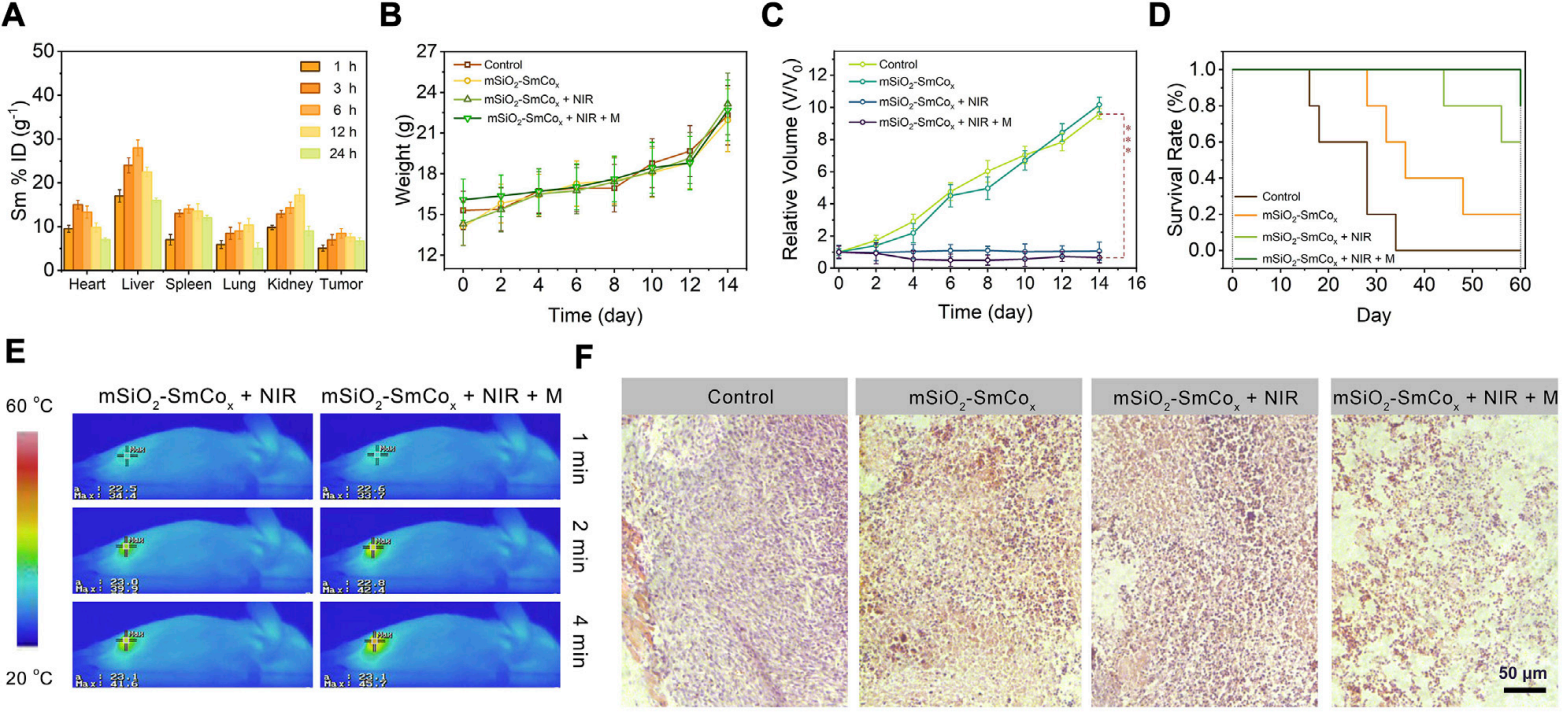
**(A)** Biodistribution of Sm ions (% injected dose (ID) of Sm per Gram of tissues) in main tissues and tumor in 1, 3, 6, 12, and 24 h of intravenous administrations of mSiO_2_-SmCo_x_ (Sm/Co = 1:2) NPs (*n* = 3). **(B)** Changes in the average body weight, **(C)** relative tumor volume, and **(D)** survival rates of mice with various treatments. **(E)** Temperature elevation at the tumor sites of 4T1 tumor-bearing mice under 808 nm laser (1.0 W cm^–2^) irradiation and 808 nm laser irradiation in magnetic conditions with mSiO_2_-SmCo_x_ (Sm/Co = 1:2) NPs for 4 min **(F)** H&E-stained photographs of tumor slices obtained from tumor-bearing mice after treatments. Error bars are based on the standard errors of the mean. Statistical analysis is assessed by unpaired Student’s two-sided t-test. ***p < 0.001, **p < 0.01, or *p < 0.05.

Considering the *in vitro* high anti-tumor efficiency of mSiO_2_-SmCo_x_ (Sm/Co = 1:2) NPs, we injected mSiO_2_-SmCo_x_ (Sm/Co = 1:2) NPs into 4T1-bearing female BALB/c mice to evaluate the further *in vivo* antitumor therapeutic performance. Firstly, to ensure biosafety before the therapeutic process, the biological distribution of mSiO_2_-SmCo_x_ (Sm/Co = 1:2) NPs after entering the tumor and organisms were estimated. For confirmed periods, the 4T1 tumor-bearing mice were euthanized after intravenous administrated, then collected the tumor and five main organs for investigating Sm ions concentration to build the biodistribution *via* ICP-OES analysis (Figure 6A). As being uptaken by the reticuloendothelial system, the mSiO_2_-SmCo_x_ (Sm/Co = 1:2) NPs mainly enrich in the liver and spleen. The accumulation of mSiO_2_-SmCo_x_ (Sm/Co = 1:2) NPs in tumors are due to the enhanced permeability and retention effect (EPR) effect, where a maximum tumor uptake of the administration around 6 h, demonstrating that mSiO_2_-SmCo_x_ (Sm/Co = 1:2) NPs exhibit a good tumor-targeted administration. Next, the enhanced anticancer effect of mSiO_2_-SmCo_x_ (Sm/Co = 1:2) NPs was explored according to the standard treatment process. When the tumor volume reached nearly 30 mm^3^, the 4T1 tumor-bearing mice were divided into four groups randomly (*n* = 5 per group): 1) control (PBS), 2) mSiO_2_-SmCo_x_ (Sm/Co = 1:2) NPs, 3) mSiO_2_-SmCo_x_ (Sm/Co = 1:2) NPs + NIR (1.0 W cm^−2^, 10 min), and 4) mSiO_2_-SmCo_x_ (Sm/Co = 1:4) NPs + NIR (1.0 W cm^−2^, 10 min) + M (AFM). mSiO_2_-SmCo_x_ (Sm/Co = 1:2) NPs were injected intravenously into each group on 1, 7, and 14 days. All the samples were exposed to NIR irradiation or magnetic conditions after 6 h intravenously injection. The tumor volume and weight of each sample were measured and recorded every 2 days throughout the whole therapy. Moreover, there was no significant weight difference among the groups of mice after 14 days (Figure 6B). As exhibited in Figure 6C, the relative tumor volume displays a trend of differentiation. The tumor growth in the 3) and 4) groups are all suppressed, and the mSiO_2_-SmCo_x_ (Sm/Co = 1:2) NPs + NIR + M group shows the most significant inhibitory effect on tumor progression, illustrating a satisfactory therapeutic effect. Comparatively, the 4T1 tumor-bearing mice still survive after 60 days of treatment in the mSiO_2_-SmCo_x_ (Sm/Co = 1:2) NPs + NIR + M group with markedly prolonged lifetime, confirming again the superior anticancer efficacy (Figure 6B). As displayed in Figure 6E, the infrared thermal images of the tumor site were acquired at different times, where the skin temperature of the 3) and 4) groups increased notably, demonstrating the excellent magnetic and photothermal effect of mSiO_2_-SmCo_x_ (Sm/Co = 1:2) NPs. The hematoxylin and eosin (H&E) staining photographs of the prepared tumor section show that the most obvious apoptosis of tumor cells occurs in the mSiO_2_-SmCo_x_ (Sm/Co = 1:2) NPs + NIR + M group (Figure 6F).

## 4 Conclusion

In summary, we demonstrated the superior magnetic/photothermal effect-induced hyperthermia therapy of mSiO_2_-SmCo_x_ (Sm/Co = 1:2) NPs to regard as an advanced synergistic hyperthermia therapeutic paradigm. The well-dispersive mSiO_2_-SmCo_x_ NPs were rationally constructed through a one-step procedure among the regulated theoretical molar ratio of Sm/Co among 1:1, 1:2, and 1:4 for controlling the dispersion and magnetism properties of SmCo_x_ NPs *in situ* growth in the pore structure of mSiO_2_. The diverse porous structures and high specific surface of mSiO_2_ areas were utilized for locating the permanent magnetic SmCo_x_ NPs. The mSiO_2_-SmCo_x_ (Sm/Co = 1:2) NPs with highly dispersed morphology have an average diameter of ∼73.08 nm and the photothermal conversion efficiency was determined to be nearly 41%. The *in vitro* and *in vivo* anti-tumor evaluation of mSiO_2_-SmCo_x_ (Sm/Co = 1:2) NPs demonstrated the promising potential for hyperthermia-induced tumor therapy due to magnetic and photothermal effects.

## Data Availability

The original contributions presented in the study are included in the article/supplementary material, further inquiries can be directed to the corresponding authors.
