# Corrigendum: Tailoring mSiO_2_-SmCo_x_ nanoplatforms for magnetic/photothermal effect-induced hyperthermia therapy

**DOI:** 10.3389/fbioe.2023.1281270

**Published:** 2023-09-19

**Authors:** Xinqiang Liang, Wenting Xu, Siyi Li, Mekhrdod S. Kurboniyon, Kunying Huang, Guilan Xu, Wene Wei, Shufang Ning, Litu Zhang, Chen Wang

**Affiliations:** ^1^ Department of Research, Guangxi Medical University Cancer Hospital, Nanning, China; ^2^ College of Material Sciences and Chemical Engineering, Harbin Engineering University, Harbin, China; ^3^ National Academy of Sciences of Tajikistan, Dushanbe, Tajikistan

**Keywords:** magnetic effect, photothermal effect, permanent magnet, mSiO_2_, tumor therapy

In the published article, there was an error in Figure 5D, 6F as published. The corrected Figure 5D, 6F and its caption appear below.

**FIGURE 5:**
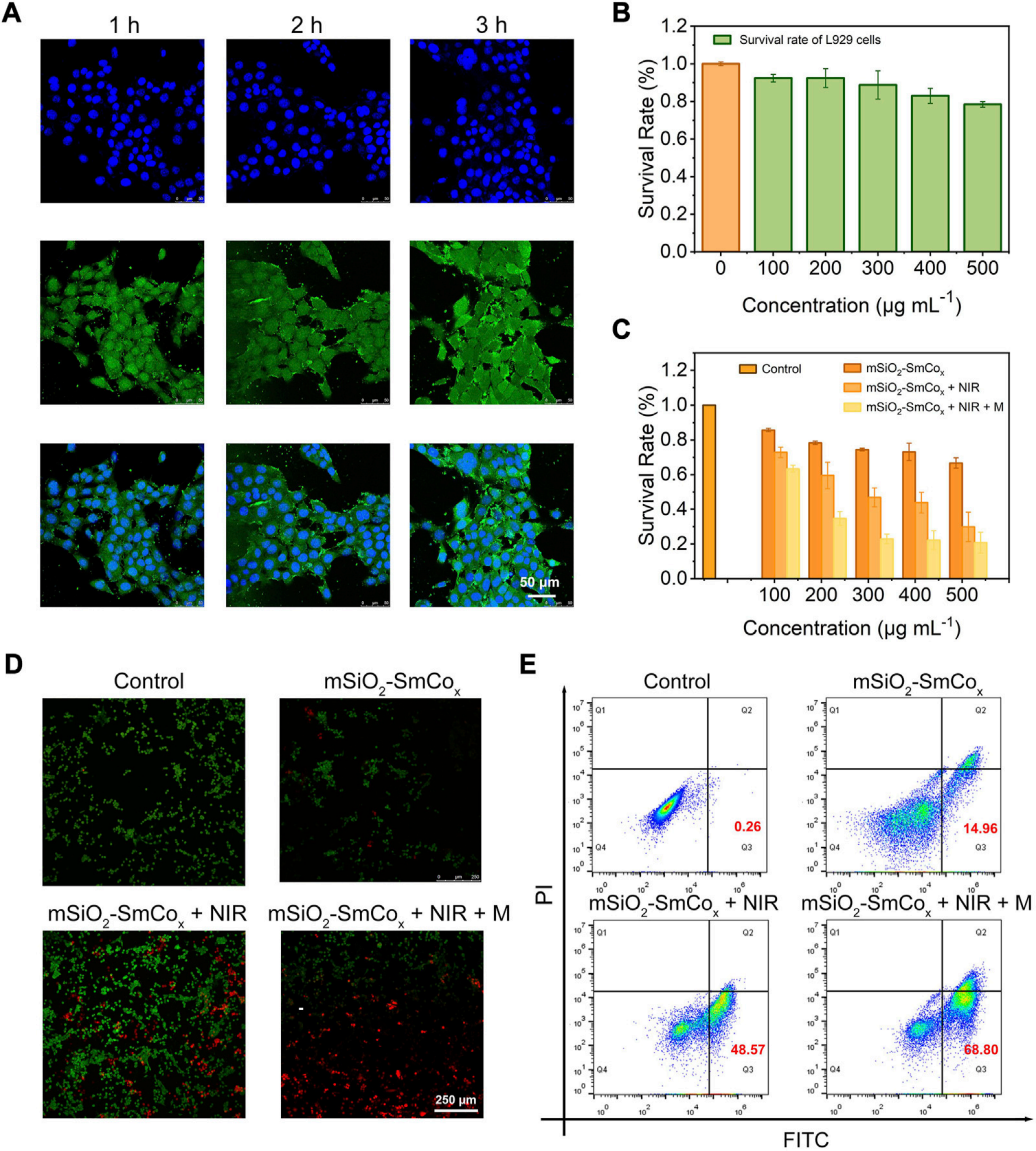
**(A)** CLSM images of 4T1 cells after coincubation with mSiO_2_-SmCo_x_ (Sm/Co = 1:2) NPs for 1, 2, and 3 h **(B)** The survival rate of L919 cells after coincubation with mSiO_2_-SmCo_x_ (Sm/Co = 1:2) NPs under different concentrations (100, 200, 300, 400, and 500 μg mL^−1^). **(C)** The survival rate of 4T1 cells after coincubation with mSiO_2_-SmCo_x_ (Sm/Co = 1:2) NPs, mSiO_2_-SmCo_x_ (Sm/Co = 1:2) NPs + NIR, mSiO_2_-SmCo_x_ (Sm/Co = 1:2) NPs + NIR + M groups under different concentrations (100, 200, 300, 400, and 500 μg mL^−1^). **(D)** AM/PI staining of 4T1 cells after different treatments. **(E)** Flow cytometry results of 4T1 cells after different treatments.

**FIGURE 6:**
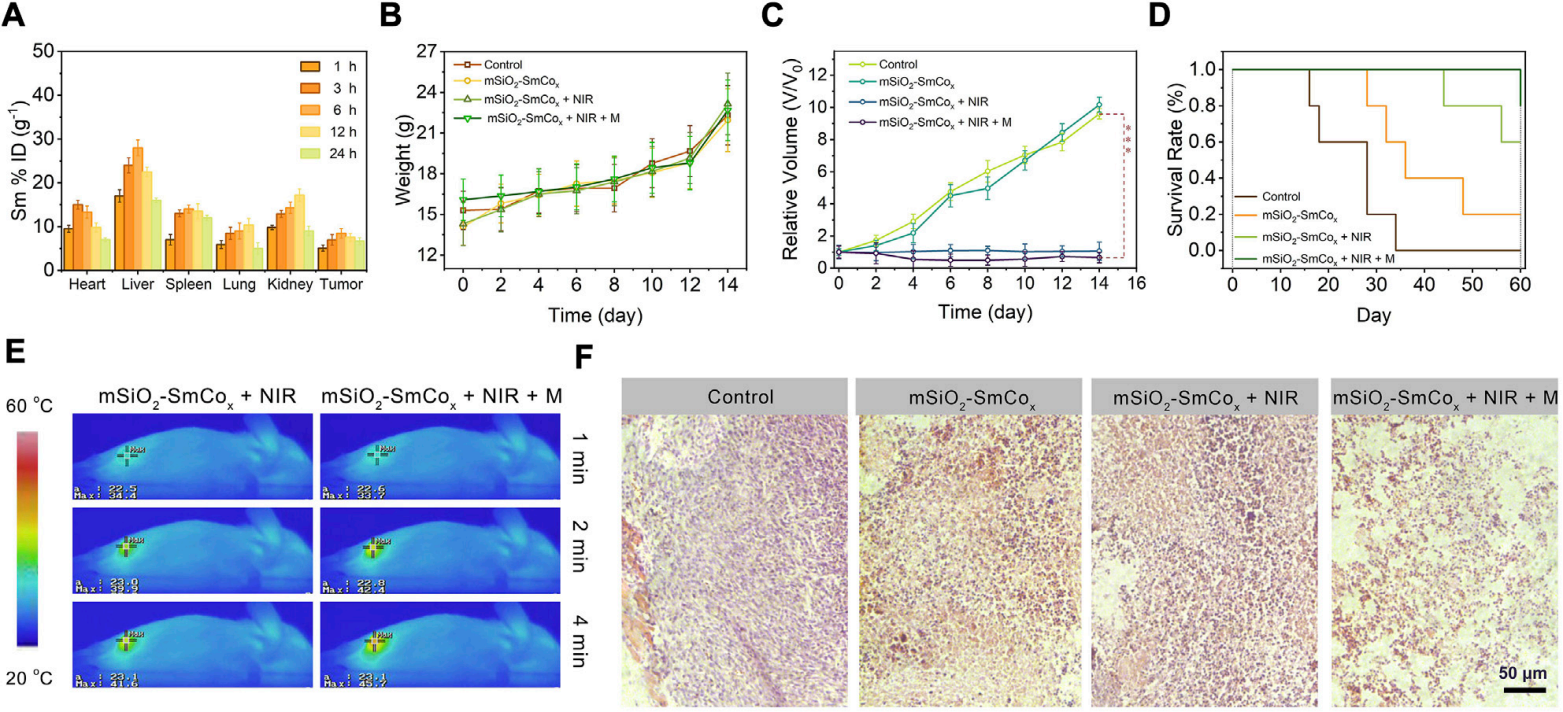
**(A)** Biodistribution of Sm ions (% injected dose (ID) of Sm per Gram of tissues) in main tissues and tumor in 1, 3, 6, 12, and 24 h of intravenous administrations of mSiO_2_-SmCo_x_ (Sm/Co = 1:2) NPs (*n* = 3). **(B)** Changes in the average body weight, **(C)** relative tumor volume, and **(D)** survival rates of mice with various treatments. **(E)** Temperature elevation at the tumor sites of 4T1 tumor-bearing mice under 808 nm laser (1.0 W cm^−2^) irradiation and 808 nm laser irradiation in magnetic conditions with mSiO_2_-SmCo_x_ (Sm/Co = 1:2) NPs for 4 min **(F)** H&E-stained photographs of tumor slices obtained from tumor-bearing mice after treatments. Error bars are based on the standard errors of the mean. Statistical analysis is assessed by unpaired Student’s two-sided *t*-test. ***p < 0.001, **p < 0.01, or *p < 0.05.

The authors apologize for this error and state that this does not change the scientific conclusions of the article in any way. The original article has been updated.

